# Routes to social prescribing outside National Health Service (NHS) structures: a systematic map

**DOI:** 10.1136/bmjph-2024-000941

**Published:** 2025-02-03

**Authors:** Emma Hazeldine, Sophie Westwood, Mohammad Hassannezhad, Stephanie Tierney, Lucy Gavens, Kerryn Husk

**Affiliations:** 1 NIHR South West Peninsula ARC, University of Plymouth, Plymouth, Devon, UK; 2 University of Plymouth, Plymouth, Devon, UK; 3 Queen Mary University of London, London, UK; 4 University of Oxford, Oxford, UK; 5 Lincolnshire County Council, Lincoln, UK

**Keywords:** Public Health, Community Health, Public Health Practice

## Abstract

**Objectives:**

Social prescribing, linking to community-based interventions to support individuals’ health and well-being, has become established across social medicine in the UK. Currently, most of the evidence and knowledge about how social prescribing pathways’ function focuses on primary care, and we know less about how social prescribing operates outside of these structures. This review explored the evidence concerning non-health service delivered social prescribing with a view to developing guidance that would support social prescribing pathways that function outside of the health service framework.

**Design:**

This paper reports a systematic mapping review of evidence concerning how community-based social prescribing pathways were delivered, exploring what these looked like, what needed to be in place for these to function, what outcomes were measured and how could non-health service pathways be supported to deliver these outcomes. The review searched database and grey sources and synthesised findings relating to how social prescribing pathways’ function.

**Setting:**

Community settings, outside of formal National Health Service (NHS) structures without statutory service input.

**Participants:**

All participants that experienced pathways were included; no limits were applied.

**Interventions:**

Non-NHS social prescribing pathways that included the core components of social prescribing.

**Main outcome measures:**

Rich descriptions of functions of pathways.

**Results:**

This mapping review included 17 studies. The synthesis indicated that NHS and non-NHS social prescribing pathways are intertwined and mutually reliant, such that it was neither sensible nor valuable to view them as separate.

**Conclusions:**

Our review provides further evidence for social prescribing as a concept, variable across all components, rather than a single, coherent model. While there exists a ‘core’ health service pathway, we suggest that further work should be done with those delivering services to understand the roles and functions that contribute but may not presently be funded.

**WHAT IS ALREADY KNOWN ON THIS TOPIC**

- There is little evidence and knowledge about non-National Health Service (NHS) routes to social prescribing.

**WHAT THIS STUDY ADDS**

- The findings of this review suggest that it is not sensible or valuable to view NHS and non-NHS social prescribing as separate because their pathways are so intertwined and connected that they are co-reliant on each other.

**HOW MIGHT THIS STUDY AFFECT RESEARCH, PRACTICE OR POLICY**

- Funding consultation should engage a broad range of stakeholders to understand the nuance and complexity of hyper local community social prescribing where non-NHS framed social prescribing pathways may make the service more accessible for some communities.

## Background

 Social prescribing, the pathway linking individuals to social interventions outside of National Health Service (NHS) healthcare for health and well-being, is the zeitgeist of social medicine in the UK and has significant political, policy and health service traction.^1^ In 2019 and again in 2023, Primary Care Networks in England received funding for at least one (additional) new link worker to deliver social prescribing.^2 3^ Link workers play a central role in social prescribing, helping people to identify their personal well-being priorities and connecting them to resources that can assist with their non-medical needs (eg, loneliness, financial worries). Link workers also play a pivotal role in social prescribing by leveraging their expertise to navigate community assets and identify tailored interventions that address individuals’ non-medical needs. Their experience ensures a holistic approach, connecting patients to appropriate local resources that enhance well-being and improve health outcomes, while generating evidence for the acceptability, reach and scope of social prescribing is complex for methodological and practical reasons as we have argued elsewhere.^4^ There is a growing evidence base relating to how patients experience referral methods^5^ and how health benefits might accrue for some groups once a referral has occurred.^6^

**Figure 1:**
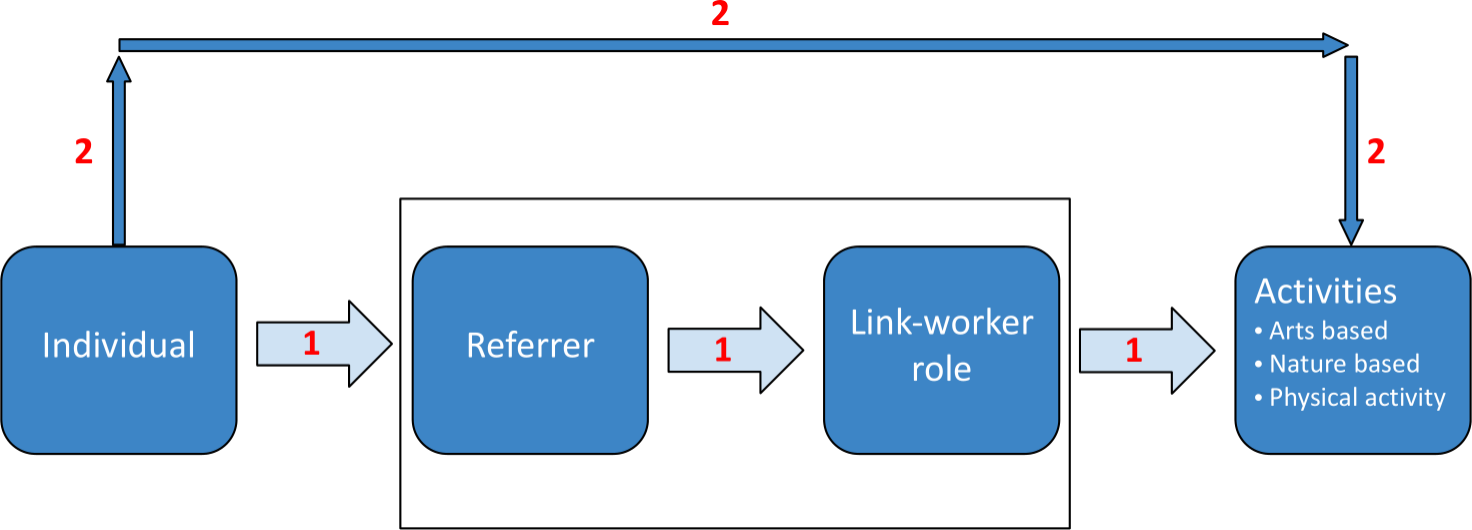
Simplified social prescribing pathways. (1) social prescribing through primary referral routes; (2) social prescribing through self-referral/community routes. Box represents health service 'scaffolding'. Adapted model from Husk *et al*.^5^

Greater clarity is required on the importance and relevance of non-NHS routes and use of community assets; however, current discussion, funding and evidence related to health-service delivered social prescribing are mostly through primary care. While we know much about how these pathways function, we must understand how these new referrals impact the community (and the voluntary and community sector) delivering activities. There is an opportunity to develop guidance to support a pathway into existing community-based activities, for which there is evidence of health and well-being effects, but without the heavy reliance on the health service ‘scaffolding’. Figure 1 illustrates how this self-referral pathway might work; however, a whole-system approach is necessary to understand how different organisations, individuals and groups can work together to function well.

In this paper, we build on our previous work in the field by presenting a systematic map of existing research and reviews focusing on community-based social prescribing offers. This review refines our understanding of what activities are offered, through which pathways, and the populations reported to be most likely to benefit. Here, we focus on post-2017 systematised/formalised approaches to social prescribing. Prior to 2017, the landscape was more diffuse, contrasting with the systematised approaches we studied. We acknowledged the practical challenges of including non-systematised, pre-2017 approaches in this review and chose to focus on the more structured models implemented after 2017 to maintain clarity and relevance.

This review maps the evidence related to how community-based social prescribing pathways are delivered. More specifically, we addressed the following questions:

1. What is the nature and variety of the routes through social prescribing pathways delivered outside more traditional NHS structures?
2. What individuals, organisations, networks and connections must be in place for these pathways?

## Methods

We conducted a systematic mapping review. Systematic mapping reviews are one of a number of approaches to summarising the evidence base for complex health topics. There are formal methods for the approach developed and published in Collaboration for Environmental Evidence (CEE), along the lines of Realist And Meta-narrative Evidence Syntheses: Evolving Standards (RAMESES) for realist reviews. This Reporting Standards for Systematic Evidence Synthesis (ROSES) approach ensures systematic rigour, making the results of mapping reviews more credible and useful for stakeholders, and we report our review in accordance with these guidelines. Mapping reviews do not aim to answer a specific research question or appraise the evidence but represent an exploratory approach to describe the nature of the evidence base, highlight gaps and identify trends.^7 8^ The process involves rigorous systematic searching and data extraction methods, with a visual and narrative synthesis of the findings. Our specific methodological approach is detailed below. We draw on the systematic mapping methodology promoted by the Collaboration for Environmental Sciences, including completing the ROSES checklist for systematic maps.^9^

### Patient and public involvement

We worked throughout with individuals in services delivering social prescribing outside of formal NHS structures and asked for direction for search terms, language and any key papers to include and to build out from. We engaged with link workers, activity providers, local authority and Voluntary Community and Social Enterprise (VCSE) organisations. Additionally, as part of our broader project work, we held multiple stakeholder consultations and interviews to gather data and build whole systems maps of these pathways, the learning from which fed into this review work. The approach to social prescribing varies across nations, with Wales (quite appropriately) adopting a more community-centred model. In Wales, link workers are deeply integrated into local communities, a practice that has been reported as more effective. However, this research focuses on social prescribing in England to maintain clarity in distinguishing between NHS and non-NHS community services, which could become blurred when comparing methods from Wales or Scotland. By concentrating on England, we can better capture variations without the added complexity of cross-national differences, ensuring a more precise and focused analysis.

### Search strategy

We undertook two main strands of searching to which we gave equal resources, in line with other systematic reviews of public health interventions detailed by Cooper *et al*.^10^ These were database searches and robust grey literature identification. ‘Our previous work (Cooper *et al*^10^) has shown that it is important to spend equal time exploring the grey literature on complex public health interventions as it is conducting formal database searches. As such, we conducted extensive grey searching for this review.

#### Database searches

We worked with an information specialist to design and refine searches for this review. We searched two databases, Scopus and Web of Science databases. During the initial consultation with topic experts, we noted no clearly defined terminology for the sorts of studies we were seeking, and defining things in the negative (ie, non-NHS) is not a helpful search strategy. As such, searching for social prescribing as a concept was most appropriate, and NHS-based studies were excluded during screening. While this increased the required screening, it was the most appropriate approach to avoid missing important studies.

We limited the search to England, as that is the context in which we apply our results; we wanted to use the results to inform our later work to develop whole system guidance for these pathways. We also limited the searches to studies published from 2017 onwards. We chose this year because the landscape of social prescribing shifted fundamentally in England then. While social prescribing existed long before that date, from then on, it saw significant policy and financial investment from the NHS. This decision also aligned with recent evidence syntheses from the National Academy for Social Prescribing.^11^ Online supplemental appendix 1 details the search strategies.

#### Grey searches

As with previous reviews on social prescribing^4 5 12^ and green social prescribing,^13^ we know that much relevant information lies outside the formal academic literature. As such, we spent significant time conducting searches of the grey literature. We took two approaches to these searches.

First, we undertook standard methods to identify studies outside of the academic literature as in previous work.^14^ We contacted known authors and experts in the field (n=12) and asked for further papers or contacts in a snowball approach; we identified and contacted relevant organisations (n=18) to ask for evaluation reports or further contacts, and we undertook Google searches (first 100 results, pasted into Word for recording) to identify websites and other organisations to contact and search. We limited these searches to English.

Our second approach to identifying relevant grey studies built on previous work by members of our team who are part of the National Academy for Social Prescribing’s Academic Partners Collaborative.^15^ This group is a collective of key research teams in the UK working on social prescribing, including the University of Oxford, University of East London, University College London, University of the West of England, The Social Prescribing Network, Sheffield Hallam University and the University of Plymouth. This group was commissioned to produce a series of evidence summaries relating to prioritised social prescribing topics. For this work, the team conducted extensive grey literature searches resulting in a cohort of evaluation reports from contacted authors, a Google search and previous work by team members. This cohort of studies was repurposed here for screening, as the projects overlapped in both timeframe and substantive focus. The academic partners collaborative permitted this to occur.

### Inclusion criteria

#### Types of studies

We included a broad range of studies which were of relevance and contributed to our understanding of how non-NHS delivered pathways function, descriptions of how potential participants are identified, the nature of the referral model, people involved in the referral model, context for referral, nature and type of support offered following referral. These sources of evidence were broad and included editorials, opinion pieces, communications, primary studies, process evaluations and systematic reviews.

#### Participants

We included studies which reported programmes including adults (>18) with a reported mental and/or physical health need that would be addressed through the pathways described above. In addition, services had to be based in England. However, given that social prescribing services operate differently for children and young people,^16^ people with dementia and individuals with autism and learning disabilities,^17^ we excluded these groups from this review.

#### Intervention and context

We included studies involving a social prescribing pathway (figure 1) that mainly functioned outside NHS structures. We included pathways involving community organisations as the referral, linking or activity providers, as well as those that included some private-sector partners such as leisure facilities.

We included self-referral to either a referral organisation, link worker or community asset directly. However, we excluded those social prescribing pathways primarily hosted by a health service, so those where referrals were made by a healthcare care professional (primary or secondary care, mental health service) or involving a primary care-based link worker.

#### Outcomes

We included any outcomes reported by included studies.

### Screening procedure and criteria

Records identified through database searches, titles and abstracts were screened independently by two reviewers (KH, EH) against inclusion criteria using Rayaan. We accessed full texts for included studies or where inclusion needed clarification. Two reviewers screened full texts (KH, EH), and disagreements were resolved through consultation with a third reviewer (ST). Studies identified through our grey literature searches were screened at the full-text stage by two reviewers (KH, SW).

### Critical appraisal

Given that this mapping review included a broad range of reports and we wanted to explicate how pathways function rather than their effectiveness, we did not undertake a formal critical appraisal of studies. This decision was in keeping with other systematic mapping reviews.

### Data extraction

Meta-data relating to included studies was extracted and organised in tabulated format to summarise the evidence base’s scale, scope and coverage. We organised data extraction around a basic social prescribing pathways framework and study characteristics such as methodological approach, sample size, date and location. Our data extraction template is found in online supplemental appendix 2.

### Synthesis

We summarised the data to describe the evidence base, narratively describing studies, the evidence scale and scope and any evidence gaps. Data from included studies were read, coded and discussed by two reviewers (KH, SW) and checked and discussed with a third (EH). We brought these studies together below using two main frameworks. First, we have reported non-NHS social prescribing programme information according to an overarching model of social prescribing used by our team in previous work (figure 1); this framework has helped us to understand social prescribing implementation in UK programmes^18^ in an international context^19^ and in working through national policy questions.^13^ In order to structure our reporting, we have presented outcome information according to the social prescribing Common Outcomes Framework we have used in previous policy work.^20^ This outcomes framework poses three key domains: impacts on the individual, on services, and on communities. Online supplemental appendix 3 details our screening synthesis.

## Results

### Search results

**Figure 2:**
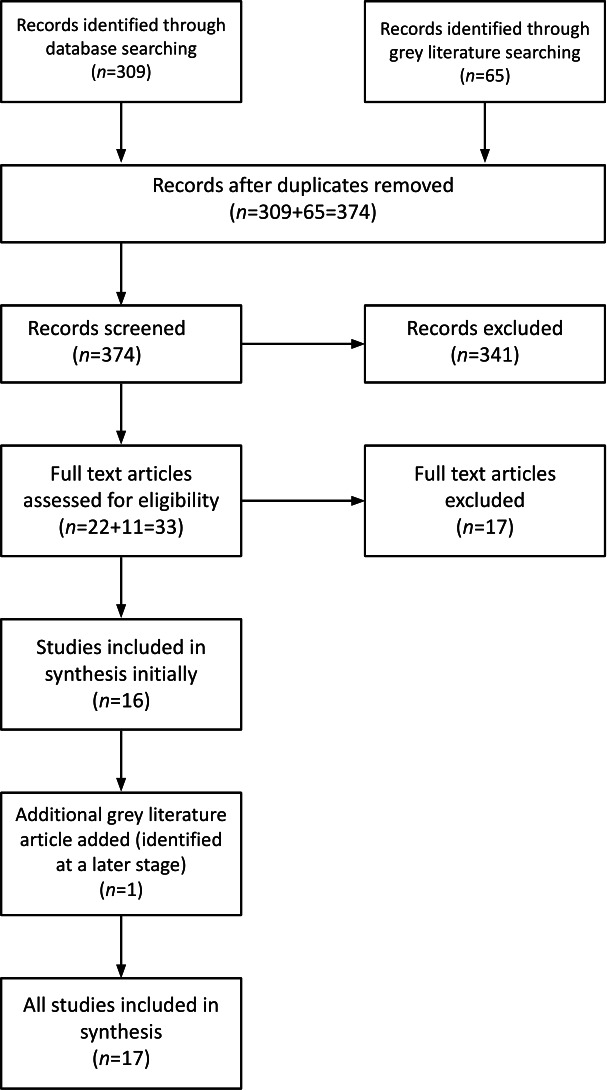
Preferred Reporting Items for Systematic Reviews and Meta-Analyses flow diagram.

We screened 374 studies, and from this cohort, 15 studies reported in 17 papers met our inclusion criteria (see Preferred Reporting Items for Systematic Reviews and Meta-Analyses diagram, figure 2). We did not locate any studies that were solely outside of the NHS. Of these 15 studies, one was a mixed methods study, two were qualitative studies, six were evaluations or reports to funders and six were programme descriptions or other documents relating to programmes. The evaluations, reports and programme documents mostly reported mixed methods also.

We applied a UK limit to our searches but located only papers relating to England. Of those papers, most related to south-east or London programmes. All included papers reported on programmes located in urban settings.

### Synthesis

#### Individual

All of the included studies were urban-based, and the majority were in the south-east of England/London based. Most studies contained a mix of methods. Sample sizes ranged from 8 participants for qualitative data collection to over 2000 data points used in quantitative analyses. Most studies had around 25 participants affording detailed qualitative data.

There may be an overlap of participants involved in more than one social prescribing programme included in these studies; we found several evaluations of initiatives in a relatively small local area with a well-established health centre. Seven of the 15 included studies ran from one established centre in a London Borough.^21^,^26^ In general, cohorts of participants were specified at the programme proposal stage. For example, a social prescribing programme might be concerned with people with a particular mental and/or physical distress, such as cancer patients,^26^ people who had been bereaved or were lonely.^23 24 27^ Sometimes studies targeted socioeconomically deprived communities or people with financial difficulties.^21^,^2628^ At the same time, due to the practicalities of delivering a programme, these populations were also place-based, that is, the populations reported in these studies were engaged in programmes that were local to and embedded in the communities that the programmes served.

Participants in the studies included a broad range of ages; nonetheless, even in studies that included young people, most participants were over 55. Four included papers reported that their service users were predominantly older women. For example, Baldwin *et al*^21^ described a service user cohort of predominantly Bangladeshi women 60 years and older who had accessed a project to support money management. Knight-Markiegi^27^ reported the evaluation of a project aimed at reducing social isolation, which engaged mainly women 80 years of age and older. Several programmes were specifically designed for older people, such as day centre programmes^29^ and a programme targeting well-being of older people in an East London Borough.^22^ The ethnicity of participants usually reflected what occurred in the study’s community. Hence, programmes run by a well-established health centre located in a socioeconomically deprived area of London reported findings from a project predominantly attended by women from the Bangladeshi community.^21^

#### Referrer

Although when we accessed the studies that reported on ‘non-NHS’ social prescribing models, when we examined studies in detail, the referrals that were detailed in the studies frequently referred via multiple routes into social prescribing service including some NHS routes. We talk later about the impossibility of treating NHS and non-NHS social prescribing as separate entities when they are in fact complex and intertwined. The studies included referrals through primary care, secondary care, other health professional services, local authority and other statutory services, VCSE, health navigators, the internet, self-referral and marketing. Often participants of social prescribing programmes in the included studies were signposted and/or linked via their general practitioner (GP). The social prescriber role could be an NHS-employed link worker, a VCSE-employed link worker or an employee with an outreach focus. Health navigator was another role cited that bridged the gap between NHS and VCSE services.

Overall, referrers were one or more of:

1. NHS and blurred NHS services.
2. Charity routes and VCSE (including outreach methods).
3. Signposting.
4. Self-referral: to an organisation, link worker and activity.

The distinction between NHS and non-NHS services is blurred, and we can see a spectrum of means of referral to social prescribing programmes that range from entirely within and entirely outside NHS structures with various hybrid pathways in between. For example, one social prescribing programme in London reported a breadth of NHS and non-NHS sources, with 45% of referrals coming from GPs, 28% from other health and social care organisations, 12% from the voluntary sector, 10% from health navigators and 5% from self-referrals.^30^ Similarly, a day centre programme to tackle loneliness accepted referrals from any source, including statutory services and self-referral.^29^ Word of mouth was mentioned in one study alongside self-referrals.

**Figure 3:**
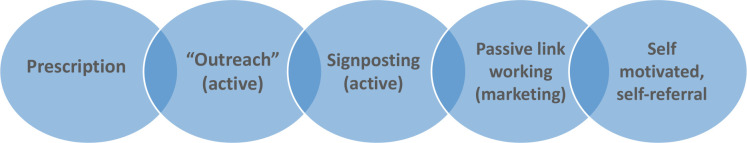
Social prescribing referral pathways.

We think these referral pathways operate on a continuum from ‘prescribed/ordered’ activity through to the self-motivated self-referral (figure 3).

#### Linking function

Commonly, a social prescribing programme includes a formal role of a link worker. A Primary Care Network often employs them; however, they can also be located in the fire service, for example, or employed in the VCSE sector, such as by a charity with specific social prescribing services. Link workers are important for cultivating ‘strong’ connections between those that refer and services that provide activities or support that form part of a social prescription. Beardmore^31^ described the importance of social prescribing staff working as part of a team, particularly when the service was co-located within the NHS. Garner-Purkis *et al*^28^ described health mentors who met with mainly self-referral clients and supported them in developing activity plans, taking baseline measures and supporting engagement with chosen activities. Where there is no link worker, a client is referred or may have self-referred directly to an activity. However, this connection is comparatively weak, with little communication between the activity and the referrer to review progress, overcome challenges and identify needs.

The internet and marketing are reported separately to self-referrals and could be classified as a passive linking function. Self-referrers can only self-refer with prior knowledge of the social prescribing services available, although no feedback mechanism is possible. The cohort of studies that described this did not detail clients’ pathways to participation in social prescribing services, nor the amount and depth of contacts involved. The location of referrers, link workers, linking functions and social prescribing services was not always reported.

#### Activities

Social prescribing activities broadly fitted into the zones posed by the National Academy for Social Prescribing: physical activity, nature-based, arts and culture, and debt or other advice. Activities were the best described of all the components in this cohort of studies. They often targeted particular cohorts of individuals: older people,^22 29^ those who were socially isolated^23 24 27 32^ and housing tenants.^21 25^ At the same time, geographic locations usually dictated activity programmes for practical reasons. All the studies were in urban areas, where we would anticipate a higher density of community organisations offering activities.

There was often a record of the number of individuals participating in an activity over its whole duration; however, due to flexible entry and exit to and from activities, it was difficult to accurately measure the impact of an activity over a set period. As a result, the cohort was usually in flux. There was also some evidence that individuals were able to re-enter a programme and that this flexibility may also have been necessary to the impact of the activity on that individual, but this was not well described. Often activity programmes were time-limited depending on the activity (eg, financial advice), or they were more extended programmes depending on the funding available (eg, gardening or arts groups). Furthermore, the size of groups participating in an activity simultaneously in the same space was infrequently described. Nonetheless, the group dynamic and ‘social’ element to activities was considered significant in the efficacy of programmes. For example, Garner-Purkis *et al*^28^ reported that the social element of a physical activity programme played a large part in the engagement of participants with the activity. Seeing other people with whom they could identify themselves helped them to find the physical activity environment less intimidating and more welcoming.

#### Outcomes

Unsurprisingly, there was a breadth of outcomes reported in included studies. For example, a study that worked with service users who had direct experience of mental distress reported the short form of the Clinical Outcomes in Routine Evaluation questionnaire^33^ and the short form of Warwick and Edinburgh Mental Wellbeing Score (SWEMWBS),^34^ along with qualitative data via written feedback.^23 24^ In addition to WEMWEBS,^35^ a study of day centre attendee characteristics reported Adult Social Care Outcomes Toolkit,^36^ Edmonton Frailty Scale^37^ and Practitioner Assessment of Network Type^38^ scores along with the qualitative findings from semistructured interviews with day centre attendees.^29^ Other projects reported qualitative outcomes such as a self-reported reduction in worries about money and a change in shopping habits.^21^ We have presented this split by the domains given in the Common Outcomes Framework for social prescribing^39^ :

- Individual:
  - Various quality of life measures, including WEMWBS/SWEMWBS.
  - Loneliness measures.
  - Measures related to debt.

Scores on individual measures tended to be positive, but the reliability or generalisability is in question given the likelihood of bias because organisations were evaluating their own provision, absence of control groups and inclusion of confounders. In addition, studies reported little change over time, primarily as it proved challenging to (a) keep people engaged over more extended periods in these pathways and (b) the practicalities of data collection became more challenging. One other key challenge was the assessment of ‘baseline’ scores where there were multiple time points; as described below, individuals often experienced more than one activity, repeated entry to activities, or dropped out and re-entered activity groups. Hence, baseline scores could rarely be classed as true baseline scores.

- Service-related measures

Measures that were related to the impact on services, in this case, the VCSE and related organisations, were not commonly included in studies. Most studies indicated an expectation that health service use—across multiple, myriad services—would decrease following engagement with social prescribing pathways. However, no similar reflection was hypothesised for VCSE service use, despite clear cases for resilience being an essential component of these pathways. Where social return on investment (SROI) had been calculated, the indirect effect on VCSE service use was not explicitly described in the included papers. We acknowledge the potential for bias in instances where studies were not independently conducted, particularly in the case of SROI calculations. These calculations were performed by the original study authors rather than by our research team, which may affect the objectivity of the results. However, Mead^26^ did report the impact of a social prescribing service that targeted people living with cancer and beyond on onward activities with defined social value, including access to advice, volunteering opportunities and joining social groups, physical activity classes and support groups. In the evaluation of a programme that supported service users to increase confidence and skills in money management, Baldwin *et al*^21^ reported that the programme’s money management activities could be embedded into other existing activities/services, which would increase engagement from participants who had not explicitly been interested in money management. However, it was not reported how that might impact VCSE service in terms of increased demand for service.

- Community measures

Measures related to the health or resilience of communities were not reported in included studies, despite being frequently mentioned (ie, ‘the health of communities’ being important to pathways’ function). Some softer, related outcomes were described in qualitative studies, for example, reports of participants ‘feeling connected’ following activities. Clift and Bungay^23^ described participants who felt they had reconnected with their community and experienced increased social integration through time spent in a therapeutic horticulture project.

## Discussion

At the outset of this review, we anticipated being able to distinguish, separate, and analyse NHS and non-NHS social prescribing routes in isolation. We felt that the commentary around alternative routes to social prescriptions was sufficiently coherent that non-NHS routes stood apart for the most part. However, the evidence in our 14 included studies shows that it was not necessarily possible, or sensible, to look at NHS and other routes separately; the overlap between them makes pathways challenging to distinguish or disentangle.

Broad referral entry points were described in keeping with findings from the social prescribing literature more broadly.^40^,^42^ Importantly, these were blended across both NHS and non-NHS services and often across elements of both in one site. There were reports of some self-referrals, but these were uncommon. These findings add weight to the notion that social prescribing is a pathway rather than a coherent entity, and each component includes significant breadth.^5 12^ These pathways also included passive link working, so along with the self-referral component, there is a continuum from ‘prescribed’ activity to self-led pathways.

The evidence bases also indicated that the role of linking and link workers are a diverse population, sitting across multiple organisations and often with multiple contracts.^17^ While it remains helpful to conceptualise this linking function as distinct for social prescribing pathways, where this function sits, how it functions and the levels of feedback to other parts of the pathway are broad and diffuse, often usefully so. This diversity in the models of linking function facilitates broader access to social prescribing activities by meeting populations where they are in community settings.

In terms of activities, the evidence base in this area indicates that people engage with activities that sit broadly across the four pillars of social prescribing articulated by the National Academy for Social Prescribing:^11^ physical activity, arts and culture, nature-based and finance, or other advice services. These activities are often targeted to particular groups. In this cohort of studies, we found a sufficient provision of activities (though with the caveat that all these studies were conducted in urban areas). Notably, activities themselves reported cohorts being in flux, constantly changing through entry and re-entry, volunteering and other feedback mechanisms.^13^

We also found a diversity of outcomes, which is common to the social prescribing literature.^6 20^ As elsewhere, we found that most outcomes related to the individual (specifically health and well-being), with a lack of measures relating to the community or service-level impacts.

### Limitations of the evidence base

Our review found that the evidence base related to non-NHS social prescribing pathways was limited in several notable ways. First, there were (quite understandable) methodological limitations in that most studies were qualitative or quantitative with no control group, comparator or multiple observations. While this enabled helpful rich descriptions of the pathways’ function to be collated, robust estimates of their operation, reach or impact were not available. While cohort studies are useful in real-world research, they may not always offer the same level of control over confounding variables, potentially making it harder to draw definitive conclusions about the efficacy of specific interventions. We would argue that a series of evidence approaches is important and multiple approaches have value, but RCTs can be designed to suit the complexities of social prescribing and potentially allow for commissioners to make funding cases where other study designs do not. Seven of the 15 studies ran from the same London centre, and most studies included were conducted (again understandably) as part of in-house evaluations and often funded by the same funder as programmes or activities. Studies were also usually conducted by existing programme staff rather than external evaluators. Participation in activities or programmes by individuals was not consistent, and data may have been skewed in that people arrived and left at different times for different periods, so any change over time was poorly estimated. Similarly, baseline estimates were often unrealistic as people may have been previous completers of programmes and may have had prior experience with activities. We acknowledge that there are limitations in the search strategy. Searching only two databases was a practical decision; however, it introduces some healthcare bias. This acknowledgement has been added to the limitations section. Using broader search terms would have made the review unmanageable to conduct and report as well as being less useful to policy and practice.

## Conclusions

Overall, we would argue that the studies included in this review indicated that it is not sensible or valuable to separate NHS and non-NHS social prescribing, given that pathways are so intertwined, connected and reliant on one another. The findings of this review give more weight to the notion that social prescribing is not one coherent ‘thing’ but an idea. This idea has been differentially interpreted and implemented across different contexts, with significant diversity and diffuseness across all components. The ‘core’ pathways are funded and delivered through NHS services, but these dovetail with and operate alongside and in support of less well-defined elements that include the VCSE, charity and other statutory services. While social prescribing indeed involves tangible and intangible approaches rooted in community development, the statement that it is not one coherent ‘thing’ but an idea highlights the diversity and flexibility inherent in its interpretation and application. Social prescribing encompasses a wide range of interventions, tailored to individual and community needs, making it less about a singular, standardised process and more about a broad, adaptable approach. While it can achieve significant public health outcomes, its strength lies in this flexibility and adaptability, which allows for different models and methods to emerge across various contexts.^43^ Therefore, it is important to recognise both the structured and evolving nature of social prescribing.

### Implications for practice

Given these findings, we would argue that there are some core areas where practice might recognise the interconnection between NHS and non-NHS services. Regarding funding, implementation and roll-out must engage a broad range of stakeholders and hold transparent, equitable conversations about development. There is some evidence that non-NHS routes might make social prescribing more accessible to certain populations, such as ethnic minority groups. One programme, embedded in the local community centre, reported their ‘typical’ participant as being female, Bangladeshi and living in social housing. Referrals into this project came from the community centre itself and partner organisations within the community.^44^ For delivery, understanding this complexity and engaging with those in local communities who understand key individuals and the new workforce around linking functions can be built into the conversations outlined above. Lastly, the system must include and encourage ‘blur’ around the boundaries of clearly defined social prescribing, working with those delivering services to explore roles and functions that contribute but may have yet to be captured or funded.

## Supplementary material

10.1136/bmjph-2024-000941 online supplemental file 1

10.1136/bmjph-2024-000941 online supplemental file 2

10.1136/bmjph-2024-000941 online supplemental file 3

10.1136/bmjph-2024-000941 online supplemental file 4

## Data Availability

All data relevant to the study are included in the article or uploaded as supplementary information.
