# Supplementary material for: Routes to social prescribing outside National Health Service (NHS) structures: a systematic map
Source: BMJ Public Health. 2025 Feb 3;3(1):e000941. doi: 10.1136/bmjph-2024-000941 (PMC11816873; doi:10.1136/bmjph-2024-000941)
Supplement: online supplemental file 1 [file bmjph-3-1-s001.pdf]

Appendix 1- systematic map searches

DATABASES

Scopus

((TITLE-ABS-KEY ("social* prescrib*") OR TITLE-ABS-KEY ("social prescription*") OR TITLE-ABS-KEY ("community referral*") OR TITLE-ABS-KEY ("social referral*") OR TITLE-ABS-KEY ("non-medical referral*") OR TITLE-ABS-KEY ("link worker*") OR TITLE-ABS-KEY ("care navigator*"))) AND PUBYEAR > 2017 AND (LIMIT-TO (AFFILCOUNTRY , "United Kingdom"))

RUN: 30.11.21 – 258 hits

WoS

TS=("social* prescrib*" OR "social prescription*" OR "community referral*" OR "social referral*" OR "non-medical referral*" OR "link worker*" OR "care navigator*")

- Languages – bibliographic: List languages to be used in bibliographic database searches.

Refined By:Countries/Regions: ENGLAND or SCOTLAND or WALES or IRELAND

Date: 2017-Present

RUN: 30.11.21- 290 hits

So 548 total into EndNote.

Duplicates = 239

TO SCREEN: 309
