# Supplementary material for: Routes to social prescribing outside National Health Service (NHS) structures: a systematic map
Source: BMJ Public Health. 2025 Feb 3;3(1):e000941. doi: 10.1136/bmjph-2024-000941 (PMC11816873; doi:10.1136/bmjph-2024-000941)
Supplement: online supplemental file 2 [file bmjph-3-1-s002.pdf]

Appendix 2 - Data extraction template

Excel column A, B, C etc	Content
A	Extracted by
B	Title/Author
C	Topic/Question
D	Methodological approach
E	Key organisations described
F	Links/infrastructure described
G	Ouputs (programme operation)
H	Outcomes (impacts on ind/comm/service)
I	Description of pathway operation
J	Progress Plus Summary
K	PROGRESS-Plus: Present? Y/N. If Y: Did SP reduce impact of characteristic? Effect of characteristic on access, acceptability, uptake?
L	Place of residence (e.g. rural/urban)
M	Race/ethnicity/ culture/language (general)
N	Info on ethnic minorities
O	Occupational group
P	Gender/sex
Q	Education
R	Socioeconomic status
S	Social connections
T	Age
U	Disability/ illness
V	Features of relationships (e.g. divorced, bereaved)
W	Time-dependent relationships (e.g. leaving hospital, retiring, new parents, starting university)
