# Supplementary material for: Routes to social prescribing outside National Health Service (NHS) structures: a systematic map
Source: BMJ Public Health. 2025 Feb 3;3(1):e000941. doi: 10.1136/bmjph-2024-000941 (PMC11816873; doi:10.1136/bmjph-2024-000941)
Supplement: online supplemental file 3 [file bmjph-3-1-s003.pdf]

Appendix 3 Results: Review, screening, and synthesis details

Results

We screened 374 studies, and from this cohort 13 studies reported in 15 papers met our inclusion criteria (see PRISMA, figure 2). Of these studies, (1/9 were mixed methods)(4/13), (2/9) were qualitative (2/13), 5/9 were evaluations or reports to funders (6/13), and (1/9) were programme descriptions or other documents relating to programmes (1/13).

We applied a UK-limit to our searches, but located only papers relating to England, and of those most related to programmes located in either the south-east or London specifically. All included papers were located in urban settings.

We bring these studies together below using two main frameworks. Firstly, we report non-NHS social prescribing programme information according to an overarching model of social prescribing used by our team in previous work, this framework has helped us understand social prescribing implementation in programmes (18), in an international context (19), and in working through national policy questions (13). We present outcome information according to the social prescribing Common Outcomes Framework which we have used in previous policy work (15). This outcomes framework poses three key domains: individual / service / community outcomes.

Model of Social Prescribing

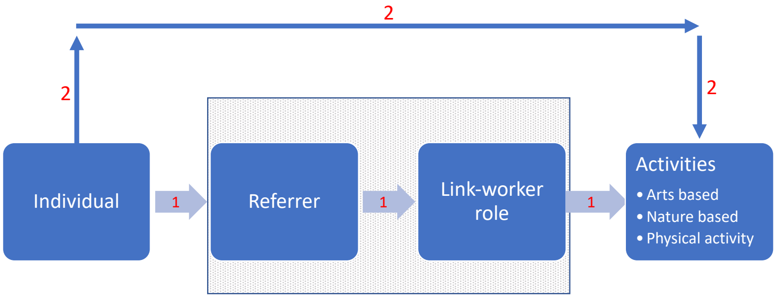

(1) – Social prescribing through primary care referral routes
(2) – Social prescribing through self-referral/community routes
Shaded box – health service 'scaffolding'

Adapted model from (Husk, Blockley et al. 2019)

The above figure represents a general model of social prescribing, and we tabulate and bring together included studies for this review based on the components of this model; to best describe the ways in which the evidence reports non-NHS social prescribing pathways to function.

i) Individual

- Cohorts / populations included
- Potential crossover between cohorts in small local areas (especially urban) – so people might be in more than one programme?
- Broad range of ages, but often project specific and targeted.
- Cohorts in general are very specific and targeted by programmes depending on aims – might be symptoms / population / SES / finance etc.
- PROGRESS-Plus here – any data relating to each group? Gaps/impacts summarised. Narrative for all.

All of the included studies were urban based and the majority in the south-east of England/London based. Most studies were mixed methods and had variable numbers of participants for different aspects of the study. Sample sizes included a range of 8 participants for qualitative data collection to over 2000 points of data used in quantitative analyses. Most studies had around 25 participants affording detailed qualitative data.

There may be overlap of participants involved in more than one social prescribing programme included in these studies because there are several evaluations of initiatives in a relatively small local area, where there is a well-established health centre. In general, cohorts of participants were specified at the programme proposal stage. For example, a social prescribing programme might be concerned with people with a particular mental and/or physical distress such as cancer patients, the bereaved or lonely. Sometimes studies target socio-economically deprived communities or people with financial difficulties. At the same time, due to the practicalities of delivering a programme, these populations were also place based.

Participants of the studies included a broad range of ages, nonetheless even in studies that included young people, the vast majority of participants were over 55 years of age and weighted more to older years than this. Several programmes were specifically designed for older people. Participants also tended to be mostly female. The ethnicity of participants usually reflected that which occurred in the community the study took place.

ii) Referrer

- Types of referral
- GP-related (dovetail to NHS services here, has to link somehow…)
- Charities having their own l/w, routes in (outreach work to source people, or self-referrals)
- Other professionals, social care, secondary care etc.
- Blur between NHS and non-NHS services (such as health navigators?)
- Internet and other 'marketing' towards services.

The studies included referrals made through primary care, secondary care, other health professional services, local authority and other statutory services, VCSE, health navigators, the internet, self-referral and marketing. Often participants of social prescribing programmes in the included studies are signposted and/or linked via their GP. This could be through an NHS employed link worker or alternatively a VCSE employed link worker, or

employee with an outreach focus. Health navigators is another role cited which bridges the gap between NHS and VCSE services. Although less represented, self-referrals are reported, and in addition referrals made through the internet and marketing are reported separately.

Overall:

i. NHS and blurred NHS services
ii. Charity routes and VCFSE (outreach methods too)
iii. Signposting
iv. Self-referral – to organisation, link worker, and activity (though these are blurred in reporting and actuality).

The distinction between NHS and non-NHS services is blurred and we can see that there is a spectrum of means of referral to social prescribing programmes that range from entirely within and entirely outside NHS structures with a variety of hybrid pathways in-between. What constitutes a self-referral is not defined and it is notable that internet and marketing referrals are reported separately. Word of mouth is mentioned in one study alongside self-referrals. Clearly, for a client to self-refer they will need to know about the service; it is not obvious that how they come about this knowledge is important to the definition of self-referral.

We think these operate on a continuum from 'prescribed/ordered' activity through to the self-motivated self-referral:

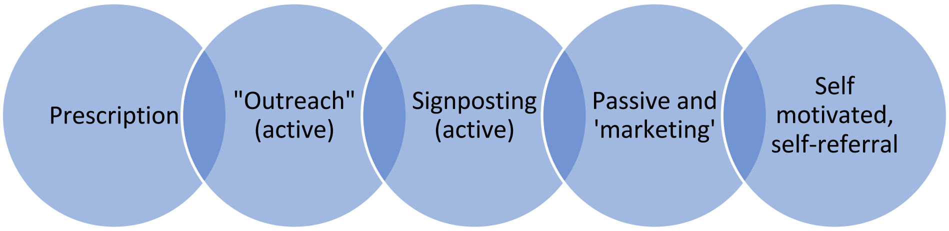

Whether a self-referring client goes through a link worker or directly to an organisation or activity is not specifically reported, and these different pathways that self-referring clients take to access social prescribing programmes create another layer of complexity.

iii) Linking function

Interested here in the 'who', the 'how' and the 'where'.

- Who:

    - Link workers as a formal role – but not all PCN etc., can be e.g., fire service, voluntary sector, charity etc. This builds a 'strong' connection.
    - No link worker – straight from referrer to activity but is a 'weak' connection.
    - Internet or marketing as the linking function – website? Database? Word of mouth?
- How:
    - Not well described in this cohort of studies, not apparent how exactly this all works.
    - Not clear the amount, type or depth of contacts.
- Where:
    - Similarly, not sure of co-location or actual place. Assumed but not reported.

A formal role of link worker is common to social prescribing programmes. They are often employed by the Primary Care Network but can also be located in the fire service for example, or employed in the VCSE sector, such as by a specific charity with specific social prescribing services to recruit for. Link workers are important to the cultivation of 'strong' connections between those that refer and the social prescribing services themselves. They also strengthen the connection between self-referring clients and social prescribing services. Where there is no link worker a client is referred, or may have self-referred, directly to an activity. However, this connection is comparatively weak, with little communication between the activity and the referrer to review progress, overcome challenges and identify needs.

The internet and marketing are reported separately to self-referrals. They could be classified as a one-way linking function for self-referrers, who can only self-refer with prior knowledge of the social prescribing services available, though no feedback mechanism is possible. This cohort of studies does not describe in detail the pathways that clients take to participation in social prescribing services, nor the amount and depth of contacts involved. Similarly, the location of referrers, link workers, linking functions and social prescribing services is not reported.

iv) Activities

- Activities are the best described out of all components in this cohort of studies
- Often targeted to particular cohorts of individuals
- But are also place-based towards communities
    - Communities in urban areas
    - Programmes are set by geography often
- 'Social' element is essential and includes a group dynamic – though size often not described.
    - Creates space and/or a safe space for group stuff
- Re-entry to programmes alluded to and important for success but needs better describing
- Flexibility of entry and exit/completion
- Ongoing and/or time limited programmes is a blurred distinction
    - Activity dependent

    - Funding dependent

Social prescribing activities largely fit into the zones posed by NASP: physical activity, nature based, arts and culture, and debt or other advice. Of all the components in this cohort of studies, activities are the best described. They are often targeted towards particular cohorts of individuals: the elderly, those who are socially isolated, housing tenants etc. At the same time activity programmes are usually set by geography for obvious practical reasons. All the studies were also based in urban areas.

The number of individuals that participate in an activity over its whole duration is often recorded but due to flexible entry and exit to and from activities, it is difficult to accurately measure the impact of an activity over a set period of time. The cohort is usually in flux. Furthermore, the size of groups participating in an activity at one time, in the same space is not often described. Nonetheless, the group dynamic and 'social' element to activities is referred to as significant in the efficacy of programmes.

Alluded to but not well described is that individuals may re-enter a programme, and this flexibility may also be important to the impact of the activity on that individual. Often activity programmes are time limited depending on the activity (for example financial advice) or longer programmes are dependent on the funding available (for example gardening or arts groups).

Outcomes

- Individual
    - QoL / WEMWBS
    - Loneliness
    - Debt
    - Often positive on all, but lots of bias and/or conflict.
    - Not much reported change over time as it was tricky to keep people engaged
    - Baseline is often confounded (see below)
    - Often seem better, or maintaining scores, but they assume low entry scores…
- Service:
    - Not commonly measured in this cohort of studies
    - Assumed decrease in health service use
    - No reflection on VCFSE use or impact – despite clear importance on resilience
- Community:
    - Not measured
    - Mentioned a lot – 'health of communities'
    - 'Feeling connected'
- Other:
    - Some SROI…

Limitations

- Methodological – most if not all were qual and associated quant studies, no controlled or comparative.
- In-house or project reports to funders – bias
- Participation was not consistent and so data may be skewed, people arrive and leave at different times for different periods – so any change over time is poorly estimated.
- Baseline is often not a realistic baseline as people may be previous completers of programmes and/or have prior experience etc.
