# Supplementary material for: Routes to social prescribing outside National Health Service (NHS) structures: a systematic map
Source: BMJ Public Health. 2025 Feb 3;3(1):e000941. doi: 10.1136/bmjph-2024-000941 (PMC11816873; doi:10.1136/bmjph-2024-000941)
Supplement: online supplemental file 4 [file bmjph-3-1-s004.pdf]

NIHR | Applied Research Collaboration South West Peninsula

MRC PHIND-SP systematic map protocol

Title:

What are the routes to social prescribing outside NHS structures? A systematic mapping review protocol

Type of review:

Systematic map

Authors:

Ms Sophie Westwood Community and Primary Care Research Group, Peninsula Medical School, Plymouth, PL6 8BX sophie.westwood@plymouth.ac.uk

Dr Emma Hazeldine NIHR Applied Research Group (ARC), South West Peninsula, Peninsula Medical School, Plymouth, PL6 8BX emma.hazeldine@plymouth.ac.uk

Dr Stephanie Tierney University of Oxford, Medical Sciences Division stephanie.tierney@phc.ox.ac.uk

Dr Mohammed Hassannezhad Department of Automatic Control and Systems Engineering, Amy Johnson Building, Portobello Street, Sheffield, S1 3JD m.hassannezhad@sheffield.ac.uk

Ms Lucy Gavens lucy.gavens@lincolnshire.gov.uk

Dr Kerryn Husk NIHR Applied Research Group (ARC), South West Peninsula, Peninsula Medical School, Plymouth, PL6 8BX kerryn.husk@plymouth.ac.uk

Background:

Social prescribing, the pathway linking individuals to social interventions outside of NHS healthcare for health and wellbeing is the zeitgeist of social medicine in the UK, and has significant political, policy, and health service traction; with each new Primary Care Network receiving funding (starting immediately) for at least one (additional) new link-worker to deliver social prescribing through NHS England. Whilst generating evidence for the acceptability, reach and scope of these pathways and activities is complex for methodological and practical reasons (as we have argued elsewhere), there is a growing evidence base relating to the ways in which patients experience referral methods[2], and how health benefits might accrue for some groups once a referral has occurred[3].

However, current discussion, funding, and evidence related to health-service delivered social prescribing is mostly through primary care. Whilst much is known about how these pathways function, little is understood about how the community (and the voluntary and community sector) who are delivering activities are impacted by these new referrals. Based on our extensive work in the field, we feel there is an opportunity to develop guidance to support a pathway into existing community-based activities, for which there is evidence of health and wellbeing effects, but without the heavy reliance on the health service 'scaffolding'. Figure 1 illustrates how this self-referral pathway might work, but in order to function well, a whole-system approach is necessary to understand how the different organisations, individuals and groups can work together.

Figure 1

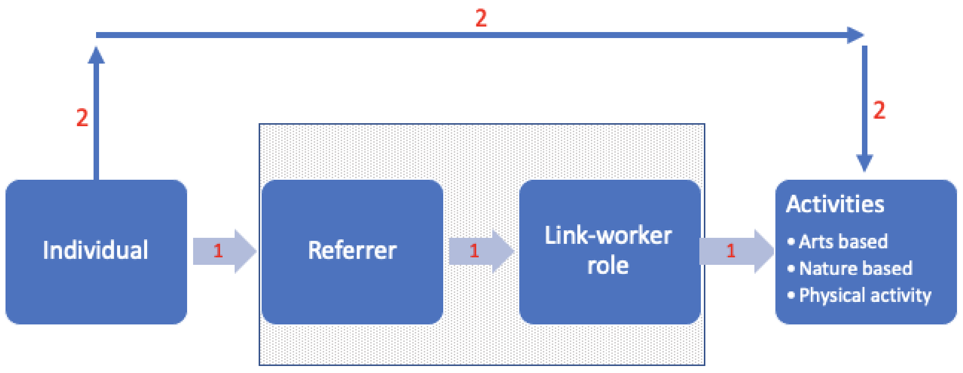

(1) – Social prescribing through primary care referral routes
(2) – Social prescribing through self-referral/community routes
Shaded box – health service 'scaffolding'

Adapted model from (Husk, Blockley et al. 2019)

We will build on our considerable previous work in the field to conduct a systematic map of existing research and reviews focusing on community-based social prescribing offers to map provision. This will refine our understanding of what activities are offered, through which pathways, and the populations

reported to be most likely to benefit. We will also identify the key organisations, individuals, and roles necessary to pathways and activities in this phase, to form the basis of a system map.

Stakeholder engagement:

Core to our approach is working with those close to these types of services. As part of the preparatory work for this review we contacted those in the field we knew had some experience, and asked for direction for search terms, language, and any key papers to include and build out from. Additionally, as part of our broader project work, we will be holding multiple stakeholder consultations and interviews to gather data and build whole systems maps of these pathways.

Objective of the review:

The aim of this review is to map the evidence as it relates to how community-based social prescribing pathways are delivered. More specifically, we will address the following questions:

1. What is the nature and variety of the routes through social prescribing pathways, delivered outside more traditional NHS structures?
2. What individuals, organisations, networks and connections have to be in place for these pathways to function?
3. What outcomes are measures for these pathways, and how can these pathways be supported and enhanced to deliver these outcomes?

Methods:

We will conduct a systematic mapping review. Mapping reviews do not aim to answer a specific research question or appraise the evidence but represent an exploratory approach to describe the nature of the evidence base, highlight gaps, and identify trends (James et al., 2016; Snilstveit et al., 2016). The process involves rigorous systematic searching and data extraction methods, with a visual and narrative synthesis of the findings. Our methodological approach is detailed below. We will draw on the systematic mapping methodology promoted by the Collaboration for Environmental Sciences, including completing the Reporting Standards for Systematic Evidence Synthesis (ROSES) checklist for systematic map protocols.

Search strategy:

In keeping with other systematic reviews of public health interventions, and in line with the approach detailed by Cooper et al. (2018), we will have two main approaches to identifying studies: database searches and robust grey literature identification.

Database searches

We worked with an information specialist to design and refine searches for this review. During initial consultation with topic experts, it became clear there was no clearly defined terminology for the sorts of studies we were seeking, and defining things in the negative (i.e. non-NHS) is not a useful search strategy. As such, we felt that searching for social prescribing as a concept was most appropriate and studies that were NHS based will be excluded during screening. Whilst this may increase the amount of

screening required, we felt it was the most appropriate approach to take in order not to miss important studies.

We will limit the search to England, as that is the context in which we want to apply our results and to inform our later work to develop whole system guidance for these pathways. We will also limit the searches to studies published from 2017 onwards. This is because the landscape relating to social prescribing shifted fundamentally in England at that time; whilst social prescribing has existed long before that date, from then on it saw significant policy and financial investment from the NHS.

Search strategies were developed with an information specialist and are given in Appendix 1.

Grey searches

As with other reviews on both social prescribing (Husk et al. 2020) and linked green social prescribing activities (Husk et al. 2016), we knew that a large proportion of relevant information would lie outside of the formal academic literature. As such we will expend significant time conducting searches of the grey literature, in line with Cooper et al. (2018).

Broadly, we will take two approaches to these searches. Firstly, we will undertake standard methods to identifying studies outside of the academic literature and as previously described in detail (Cooper et al. 2018; Lovell et al. 2016). For the reasons given above we will limit these searches to English. We will: contact known authors and experts in the field and ask for further papers or contacts in a snowball approach; we will identify and contact relevant organizations to ask for evaluation reports or further contacts, and we will undertake Google searches (first 100 results, pasted into Word for recording) to identify websites and other organizations to contact and also search these organizations' webpages where possible.

Our second approach to identifying relevant grey studies builds on previous work in this field by our team which resulted in a cohort of studies to be screened.

'NASP APC' Cohort of studies

The National Academy for Social Prescribing recently created an Academic Partners Collaborative (NASP APC). This is a group consisting of some of the key research teams in the UK working on social prescribing, including the University of Oxford, University of East London, UCL, UWE, The Social Prescribing Network, Sheffield Hallam University, and the University of Plymouth (https://socialprescribingacademy.org.uk/academic-partners-collaborative/).

This group was commissioned to produce a series of evidence summaries relating to prioritized social prescribing topics. As part of this work, the team conducted extensive grey literature searches resulting in a cohort of evaluation reports from contacted authors, a Google search, and previous work by team members This cohort of studies is repurposed here for screening, as the projects overlap in both timeframe and substantive focus. Permission was given by the APC for this to occur.

Inclusion Criteria:

Types of studies

We will include evidence which is of relevance and contributes to our understanding of how these non-NHS delivered pathways function, descriptions of how potential participants are identified, the nature of the referral model, people involved in the referral model, context for referral, nature and type of support offered following referral.

These sources of evidence are likely to include but not limited to editorials, opinion pieces, communications, primary studies, process evaluations and systematic reviews.

Participants

We will include any studies which include adults (>18) (so we will include studies which have young people as well as adults) with a reported mental and/or physical health need to be addressed through these pathways. Services have to be based in England.

Given that we know social prescribing services operate differently for children and young people (see here), those with dementia, and for those with autism and learning disabilities (Featherstone et al. 2021), we will exclude these groups from this review.

Intervention and context

We will include any study that involves a social prescribing pathway that functions in the majority outside of NHS structures. For us, this means pathways that have the four core functions of an SP pathway: individual with need, a referral function (including a self-referral), a linking function, and a community-based asset – all for the purpose of health.

We include pathways that involve community organisations as referral, linking or activity providers, as well as those that might include some private-sector partners such as leisure facilities.

We include self-referral, to either a referral organisation, link worker, or community-asset directly. There are grey areas we want to explore with our stakeholders around self-referral and more simplistic positive health behaviours, but where this is a motivated and directed attempt to address health or wellbeing using community assets, or if it is termed social prescribing, we will include. These blurred boundaries will form part of our analysis and help us better describe what works and in what ways for different cohorts.

We will exclude those social prescribing pathways that are hosted in the large part by a health service; so those where referrals are made by a healthcare care professional (primary or secondary care, mental health service), or involving a PCN/primary-care based link worker. We include a number of researchers who have spent significant time researching these pathways and will reflect on these as comparators where appropriate.

Outcomes

We will be interested in all outcomes reported by included studies. We anticipate these falling into the three domains given in the national outcomes framework, so relating to the individual (physical and

mental health, wellbeing), the community (number of volunteers, resilience of organisations), and the system (here to include non-NHS partners).

However, we will be broad in our data extraction to capture the range of impacts organisations are seeking to capture.

Screening procedure and criteria

For records identified through database searches, titles and abstracts will be screened independently by two reviewers (KH, SW), against inclusion criteria using Rayaan. Full-texts will be accessed for studies which were included or where inclusion remains unclear. These full-texts will be screened by two reviewers (KH, SW) and disagreements resolved through consultation with a third reviewer (ST).

Studies identified through our grey literature searches will be screened at full-text stage by two reviewers (KH, SW).

Critical appraisal

Given this is a mapping review and we include a broad range of reports, and are seeking to explicate the ways in which pathways function and in what ways for what groups, we will not undertake formal critical appraisal of studies. This is in keeping with other systematic mapping reviews and the guidance published.

Data extraction

Meta-data relating to included studies will be extracted and organised in tabulated format in order to summarise the scale, scope and coverage of the evidence base in this area.

We will organise data extraction of included studies around the framework given above, along with study characteristics such as methodological approach, sample size, date and location. As such, we will group data into that relating to individual need, referral function, linking function, community asset, and outcomes (themselves organised as above around the national outcome framework).

Synthesis:

Data will be organised using the frameworks given above. We will use these frameworks to summarise the evidence base, narratively describing included studies, evidence scale and scope as well as key evidence gaps.

Included quantitative studies will be tabulated and coded according to the pathway and outcome domains, with colours denoting differing organisations and – where appropriate – impacts on individuals, communities and systems. Coding will be conducted by one reviewer and checked by another.

Included qualitative and other studies will coded, using the same frameworks, in NVivo, by one reviewer and checked by another.

We will bring together these syntheses into an overall narrative synthesis describing the knowledge clusters and gaps, as well as how these data relate to relevant linked topics, such as NHS-delivered social prescribing.

APPENDIX 1 – Search Strategies

Scopus

((TITLE-ABS-KEY ("social* prescrib*") OR TITLE-ABS-KEY ("social prescription*") OR TITLE-ABS-KEY ("community referral*") OR TITLE-ABS-KEY ("social referral*") OR TITLE-ABS-KEY ("non-medical referral*") OR TITLE-ABS-KEY ("link worker*") OR TITLE-ABS-KEY ("care navigator*"))) AND PUBYEAR > 2017 AND (LIMIT-TO (AFFILCOUNTRY , "United Kingdom"))

WoS

TS=("social* prescrib*" OR "social prescription*" OR "community referral*" OR "social referral*" OR "non-medical referral*" OR "link worker*" OR "care navigator*")

- Languages – bibliographic: List languages to be used in bibliographic database searches.

Refined By:Countries/Regions: ENGLAND or SCOTLAND or WALES or IRELAND
